# Effect of a Modified Silicone as a Thickener on Rheology of Liquid CO_2_ and Its Fracturing Capacity

**DOI:** 10.3390/polym11030540

**Published:** 2019-03-21

**Authors:** Qiang Li, Yanling Wang, Fuling Wang, Qingchao Li, Forson Kobina, Hao Bai, Lin Yuan

**Affiliations:** 1 College of Petroleum Engineering, China University of Petroleum (East China), Qingdao 266580, China; B16020053@s.upc.edu.cn (Q.L.); forsonkobina@ymail.com (F.K.); S17020298@s.upc.edu.cn (H.B.); S16020312@s.upc.edu.cn (L.Y.); 2 College of Science, China University of Petroleum (East China), Qingdao 266580, China; B18090002@s.upc.edu.cn

**Keywords:** polydimethylsiloxane, CO_2_ thickener, CO_2_ fracturing technology, oil and gas development engineering

## Abstract

The low viscosity of pure liquid CO_2_ hindered the development of CO_2_ fracturing technology. A modified silicone polymer was prepared as a CO_2_ thickener to investigate the effect of temperature, pressure, shear rate and thickener content (wt.%) on the apparent viscosity and rheology of thickened liquid CO_2_. In addition, CO_2_ fracturing capacity was evaluated with the numerical simulation of extended finite element. The results displayed that an apparent viscosity of up to 1.3 mPa·s at 303 K and 18 MPa was attained over liquid CO_2_ using the thickener of 3 wt.% and Toluene of 9 wt.% as additives. Compared to the commercial linear polydimethylsiloxane, a better apparent viscosity was obtained from the mixture of this prepared thickener, Toluene and CO_2_. The apparent viscosity decreases with increasing temperature and shear rate. By contrast, an improving apparent viscosity was revealed with an increase in the pressure from 8 to 14 MPa and thickener content from 1 to 3 wt.%. The rheological index decreased with increasing thickener content with pressure but the rise in temperature led to an increasing rheological index. The mesh structure theory of the thickener, CO_2_ and Toluene molecules was in this paper gives a good explanation for the discrepancy between CO_2_ viscosity with the thickener content, temperature, pressure, or shear rate. Compared to pure CO_2_, the numerical simulation of CO_2_ fracturing demonstrated an excellent fracturing capacity by using the thickened CO_2_ fracturing fluid in shale reservoirs. This investigation could provide the basic reference for the development of CO_2_ fracturing technology.

## 1. Introduction

As greenhouse effect increases, there is much attention focused on carbon use to reduce CO_2_ emissions [1]. As an important measure to use CO_2_, CO_2_ fracturing in oil and gas exploration has been considered as an effective technology to alleviate the greenhouse effect and enhance oil production [2]. In comparison to hydraulic fracturing fluid, CO_2_ fracturing technology embodies enormous potential in protecting the environment. This will cause minimal damage to the reservoir due to its excellent backflow performance [1,3,4,5]. Low permeability shale reservoirs exhibited the same inclusiveness for hydraulic fracturing and CO_2_ fracturing, and a proppant was employed to sustain these fractures in ultra-low permeability reservoirs [6]. In hydraulic fracturing stimulation, the low permeability reservoir would be damaged owing to the blockage of chemical additives and water [7,8]. Similarly, because of the low viscosity of pure CO_2_, the effectiveness of CO_2_ fracturing is questioned. Typically, the low viscosity may show defects, which consists of the proppant settling, a poor proppant carrying ability and crack support [9]. Many attempts for improving the viscosity of CO_2_ have been conducted. However, it was also a huge challenge to screen an efficient method for improving the ability to carry proppants.

An approach to enhance the apparent viscosity of CO_2_ was to use a chemical [10,11,12,13] which can dissolve in pure CO_2_ at the constant temperature and pressure of a shale reservoir with the low permeability. In previous studies, Fluoropolymers and Hydrocarbon polymers have been of interest to scientists due to their efficient thickening performance and CO_2_ use [11,12,13]. Despite the success of these projects, polymers lead to many disadvantages which include polymer residue in the reservoir, large pressure required for fracturing, damage of the reservoir caused by large cracks and consumption of polymers by biological organism. Moreover, because of their relatively poor thickening capacity, expensive and hazardous impact on the environment, the use of Fluoropolymers and Hydrocarbon polymers as a CO_2_ thickener maybe limited. In addition to the above two polymers, siloxane polymers have been investigated as one of excellent candidates for CO_2_ thickener due to their appropriate thickening performance [14,15], higher backbone flexibility and higher CO_2_-solubility [16,17]. Moreover, as an inexpensive, environmentally benign polymer, preparation or purchase was relatively convenient. However, the main challenge for siloxane polymers is their thickening performance, large dissolution pressure and high additional amount of thickener with cosolvent [10,18]. To improve the thickening performance of siloxane polymers, many methods have been adopted, and the silicone modification has presented an outstanding ability in improving the apparent viscosity of CO_2_ [19]. To improve the solubility and interaction of polymers in CO_2_, these electron-donating groups such as the carbonyl or ether groups [20,21] could be introduced into the silicone polymer. As an electron-donating group, the epoxy group presented the same function as carbonyl and ether group_._ It should be noted that the epoxy group exhibited excellent stability in the hydrosilylation [22,23,24]. Meanwhile, the rheology of CO_2_ fracturing fluids has a critical impact on the evaluation of the fracturing property.

In this study, the Epoxy Ether-based Polydimethylsiloxane (EEPDMS) was synthesized. The effect of temperature, pressure, shear rate and EEPDMS content on the apparent viscosity of CO_2_ was investigated. A mesh structure model was proposed to explain the changing thickening performance of EEPDMS at different conditions. To compare the fracturing difference between pure CO_2_ and thickened CO_2_, this paper constructed the shale reservoir model with a low permeability, which was used in observing the CO_2_ fracturing consequent.

## 2. Materials and Methods

### 2.1. Materials

Octamethylcyclotetrasiloxane (99.9%), Tetramethyldisiloxane (99.0%) were purchased from Shenzhen Osbang New Material Co., Ltd. (Shenzhen, China). Glycidyl methacrylate (97.0%) was obtained from Aladdin Reagents (Shanghai, China) Co., Ltd. Other chemicals (Ethanol (99.0%), Toluene (99.5%), Sulfuric acid (98.5%) and Chloroplatinic acid (>99.9%)) were procured from Sinopharm Chemical Reagent Co., Ltd. (Shanghai, China). In this synthesis experiment, all drugs were sampled as received unless otherwise without further purification.

### 2.2. Preparation of EEPDMS

**Figure 1:**
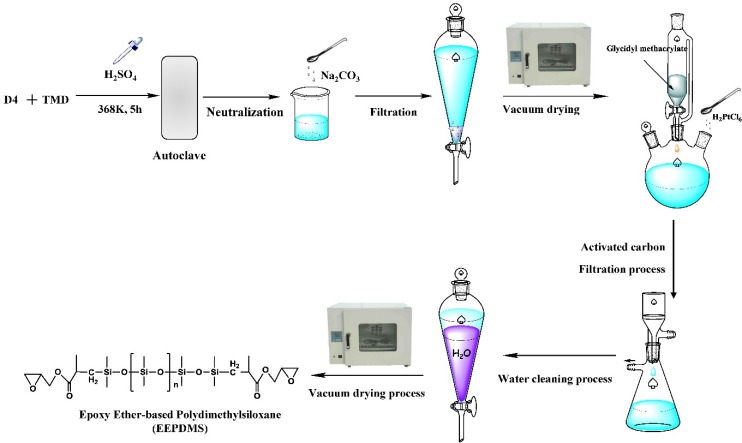
The synthesis process of EEPDMS.

All chemicals and solvents were sampled as received unless otherwise without further purification and were stored at 278 K and anaerobic environment. For the synthesis of EEPDMS, typical procedures were as follows (Figure 1). At first, Ring opening polymerization [25] was performed in a 500 mL autoclave. 60 g octamethylcyclotetrasiloxane (D4), 5 g Tetramethyldisiloxane (TMD) and 0.2 g sulfuric acid were added to an autoclave. After flushing the autoclave 30 min with N_2_, the autoclave was closed and put in a roller furnace. Then temperature was controlled to 368K, and stirring operation was conducted by the rotation of the closed autoclave in this roller furnace. After reacting for 5 h, the autoclave was cooled to 298 K. 1 g sodium carbonate was used to remove the sulfuric acid. To remove water and low boilers, the primary product was transferred into a vacuum drying oven at 393 K and 0.09 MPa. Secondly, the pure Hydrogen terminated polydimethylsiloxane (primary product) was poured into a three-necked flask, and hydrosilylation [26] was started with addition of Glycidyl methacrylate (GM) of 4.1 g and Chloroplatinic acid (0.005 wt.%) at 373 K for 3 h. To remove chloroplatinic acid, 2 g activated carbon was added into this secondary product, and then the mixture was purified by repeated-washing by using the distilled water after stopping the hydrosilylation. Water and small molecule compounds were removed by a rotary evaporator at 370 K and a vacuum of 0.06 MPa. The final product was stored in a sealed container under a low temperature (280 K). EEPDMS molecular weight of 21,000 (Mη) was measured by an ubbelohde viscometer [27,28]. It should be noted that the epoxy group was not to be destroyed, and many investigations have been proved this conclusion [22,23,24].

### 2.3. EEPDMS Characterization

Fourier transform infrared spectroscopy (FT-IR) spectrum was carried out by a Bruker Tensor 27 (Berlin, Germany) equipped with a DLaTGS detector. EEPDMS was smeared on a KBr pill and then scanned from 450 cm^−1^ to 3500 cm^−1^ with a resolution of 2 cm^−1^. In addition, the nuclear magnetic resonance spectroscopy (^1^H-NMR) was conducted using a Bruker AVANCE III (Berlin, Germany) at a frequency of 400 MHz. Deuterated chloroform was taken as the solvent, and 0.05 mL of EEPDMS was dissolved in 0.15 mL deuterated chloroform for 30 min.

### 2.4. Measurement Device and Calculation Method

**Figure 2:**
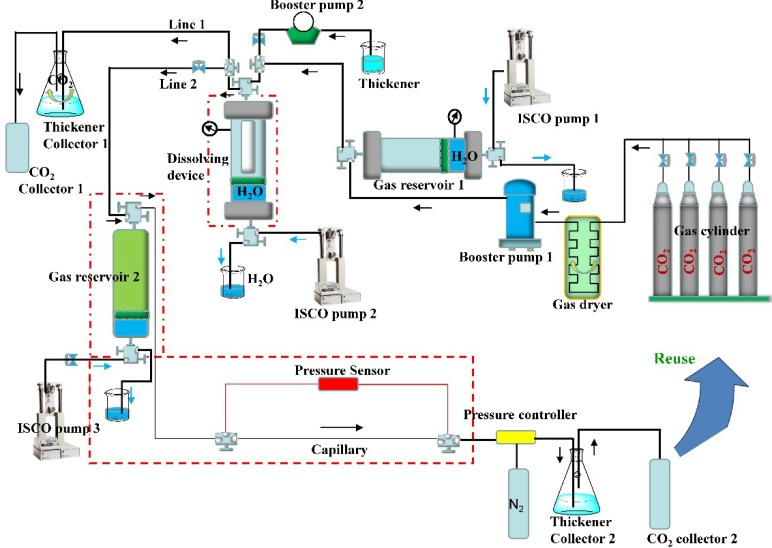
The composition of this capillary viscometer.

As shown in Figure 2, this investigation was conducted in a self-designed viscosity measuring device, which included a CO_2_ pressurization system, pressure fine-tuning system, dissolution measurement system, viscosity measurement system, and data acquisition system. This measurement system not only was employed in measuring the viscosity and rheology of thickened CO_2_, but also obtaining the solubility of EEPDMS in CO_2_ by observing the appearance of the solution.

The booster pump 1 shown in Figure 2 was used in pressurizing and liquefying CO_2_. The preliminary pressurized liquid CO_2_ was stored in the gas reservoir 1 (6 L, radius of 120 mm) immediately fabricated from the stainless steel. A gas dryer, installed in front of booster pump 1, was employed in absorbing moisture from CO_2_. To add EEPDMS to the dissolving device, a booster pump 2 was designed. Dissolving device with a visible window was a core part of this apparatus, which was used to observe the appearance of thickened liquid CO_2_ at different pressures and temperatures. Here, the appearance of thickened liquid CO_2_ mainly consisted of the insoluble state, turbid state, and clarified state. Only the thickened CO_2_ with a clarified state was pressured into the viscosity measurement system, which included a gas reservoir 2 and capillary (radius of 0.004 mm, length of 5 m). By contrast, other CO_2_ with the insoluble state or turbid state would be exhausted through the line 1. The ISCO pump 3 displayed schematically in Figure 2 was used in imposing a stable flow rate of the thickened liquid CO_2_ in the capillary. Hagen–Poiseuille equation was used to calculate the liquid viscosity according to the pressure difference and capillary parameters.

Moreover, The relationship between consistency coefficient *K* and Rheological index *n* can be expressed by Equation (1) [9].

(1) $$\mathrm{lg}\tau_{w}=\mathrm{lg}K{\left(\frac{3n+1}{4n}\right)}^{n}+n\mathrm{lg}(\frac{8v}{D})$$

where 8*v*/*D* is the apparent shear rate, s^−1^, and *τ_w_* is the wall shear stress, Pa. *v* is the flow rate, m·s^−1^, *D* is the capillary diameter, m. It could be seen that there was a linear relationship was displayed between Log*τ_w_* and Log(8*v*/*D*) herein. *n* is the slope of the straight line, and the Log${\left(\frac{3n+1}{4n}\right)}^{n}$ was considered as the intercept. *K* could be calculated by the intercept value and obtained *n* value.

### 2.5. Cosolvent Screening

The screened solvents with different polarities were mixed with EEPDMS to obtain a mixed liquid and placed at a sealed glass vial. The rotary viscometer, operated at room temperature, was used to measure the solution viscosity. In addition, the solubility and phase behavior of EEPDMS in organic solvents was determined via observing the absorbance variation gauged by a UV-visible spectrophotometer [29].

### 2.6. Phase Behavior for the Thickened Liquid CO_2_

The phase behavior and solubility of EEPDMS in solvent/CO_2_ mixtures were measured by using a variable-volume view cell, which is equipped with the pressure-resistant glass window (Figure 2). Here, the phase behavior of the mixture was observed with the judgment to know if there was an excellent solubility of EEPDMS in CO_2_ at the given temperature and pressure. Generally, an excellent solubility is prerequisite for further measurement of the thickening performance of EEPDMS in solvent/CO_2_ mixtures [30].

The capillary viscometer mentioned previously was used to assess the viscosity of solution included EEPDMS, solvent, and CO_2_. The influence of different factors on the viscosity and rheology of thickened liquid CO_2_ was discussed and interpreted emphatically.

### 2.7. Numerical Modeling and CO_2_ Fracturing Simulation

**Figure 3:**
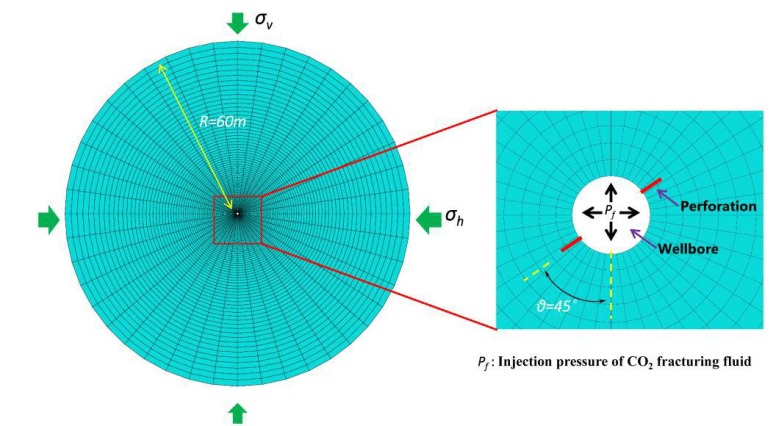
Schematic diagram of the established finite element model.

Many attempts to simulate the fracturing process mostly focused on the hydraulic fracturing technology, but there also was a limitation in the study on the initiation and propagation of the CO_2_-induced fractures in fracturing operation. Given that, the extended finite element method (XFEM), developed from the finite element model (FEM), was used in simulating fractures change in CO_2_ fracturing process. This fracturing model established here assumed that CO_2_ fracturing operation was quasi-static and stable, and the shale reservoir was treated as the homogeneous isotropic linear elastic material. Meanwhile, physical parameters of shale reservoirs were not impacted with changing stresses during CO_2_ fracturing process. Furthermore, a CO_2_ fracturing model with a large enough size (60 m) was constructed where the boundary conditions were not affected by the CO_2_ fracturing process. For convenient comparison with the fracture effect difference between the pure CO_2_ and thickened CO_2_ fracturing fluid, the viscosity of the fracturing fluid was assumed to be stable consistently. In addition, the CO_2_ fracturing simulation with the directional fracturing technology applied in the shale reservoirs, was treated as a plane strain problem [31]. As shown in Figure 3, the 2D mesh model with 15,000 CPE4P elements we establish could be used to the CO_2_ fracturing simulation with the oriented perforation. To improve the model convergence and results accuracy [31,32], the mesh refinement was conducted, especially in the near-wellbore formation. Meanwhile, the established model did not only fix the normal displacement of the wellbore and the outer boundary of the model, but also defined an outer boundary with a fixed pressure which was equal to the initial pore pressure. The reservoir properties of the model we established and fracturing construction parameters, as an extremely important foundation of CO_2_ fracturing simulation, were presented in Table 1. The two sides of the wellbore established perforations along 45° and the perforation depth was 0.15 m. The ABAQUS software was used to simulate influences of the fracturing fluid viscosity and physical parameters of the shale reservoir on the width and length of oriented crack. It should be noted that the CO_2_ fracturing fluid was injected into the perforation edged the wellbore. The investigation also explored the filtration coefficient in the shale reservoir during CO_2_ fracturing, and a filtration coefficient below 1 × 10^−14^ m^3^·(s·Pa)^−1^ at the 0.05 of porosity and 4 × 10^−17^ *K*/m^2^ of permeability was shown, which showed that the effect of CO_2_ filtration on the fracturing property was negligible.

## 3. Results and Discussion

### 3.1. Structural Characterization of EEPDMS

The synthesis and post-modifications of polydimethylsiloxane, namely EEPDMS, was monitored by infrared spectroscopy (see Supplementary, Figure S1). It can be seen from the infrared spectroscopy that the sharp peak at 2972 and 2883 cm^−1^ correspond to the C–H stretching vibration, and the peak at 1740 cm^−1^ can be attributable to the C=O bending vibration [33]. The sharp peak observed 1416 cm^−1^ indicated the asymmetric vibration of Si–CH_3_. In addition, the narrow peak of 1170 cm^−1^ (obstructed by the Si–O–Si peak at 1021–1200 cm^−1^) and 1018 cm^−1^ indicated the stretching vibration of C–O–C group and the epoxy group, respectively. The absorption band occurring between 1090 ± 1020 cm^−1^ indicated the symmetrical stretching vibration of the Si–O–Si [18]. Meanwhile, the peak at 1264 cm^−1^, attributable to the symmetrical vibration of Si–CH_3_ and CH_3_, and the peak at 800 cm^−1^ confirmed the presence of the telescopic vibration of Si–C.

^1^H NMR was used to verify the chemical structure of EEPDMS (see Supplementary, Figure S2). ^1^H-NMR (400 MHz, CDCl_3_), δ (ppm), 0.11–0.28 (m, 516 H), 0.69 (m, 2 H), 1.19 (m, 6H), 1.89 (s, 2H), 3.86 (m, 2H), 4.34 (d, 2H), 3.17 (s, 2H), 2.54 (s, 2H), 2.79 (s, 2H), 7.39(s, CDCl_3_). The FTIR spectroscopy and ^1^H NMR spectroscopy demonstrated that EEPDMS is pure (100 wt.%), and there are no impurities.

### 3.2. Solubility and Phase Behavior of EEPDMS in Organic Solvents

Due to the lower solubility of siloxane in liquid CO_2_, the solubility and viscosity-increasing property of EEPDMS in organic solvents that considered as a cosolvent in liquid CO_2_, was a basis for further measurement of the solubility and thickening property of EEPDMS. EEPDMS was evaluated for viscosity and phase behavior in organic liquids at contents ranging from 1 to 4 wt.% and temperatures at the range of 283 to 323 K employing an ubbelohde viscometer and a UV-visible spectrophotometer respectively, and the results is shown in Table 2.

Moreover, Table 2 also listed the solubility of EEPDMS in organic solvents. The appearance of mixture composed of EEPDMS and each organic solvent was significantly varied, from transparent liquid to turbid solution. For the Toluene, Hexane, and Pentane, a clear and viscous liquid was always observed with increasing EEPDMS content and temperature. The exception of these above organic liquids which was classified as a fluid with strong polarities were measured to be insoluble with EEPDMS.

Toluene or Hexane with a low content EEPDMS illustrated a medium viscosity at a low temperature. Meanwhile, the decreased viscosity gradually with an increase in temperature between 283 and 323 K, attributable to a decreased intermolecular interaction and the larger intermolecular distance microscopically [34], showed the decrease in solubility and viscosity of EEPDMS at the high temperature. However, for the Pentane with 1 wt.% EEPDMS, a medium viscosity was shown at low temperature, but the temperature range of the medium viscosity is shorter than that of Toluene and hexane. Changing the EEPDMS content to 4 wt.% obviously increased the viscosity of the Pentane, as displayed in Table 2. The drastic difference in the measured viscosity of Pentane containing different EEPDMS contents was likely due to the increased intermolecular interaction when EEPDMS molecules were raised per unit volume. In comparison to Toluene and hexane, Pentane still showed a poor thickening organic solvent and miscibility, and it will not be considered as a solvent of EEPDMS in CO_2_. By contrast, a turbid state was shown when alcohol solvents were used as a solvent and EEPDMS could not dissolve in an alcohol solution. This is mainly because there was a large difference in the solubility parameter between siloxane and these alcohol solvents [35,36,37].

Given that, Toluene and Hexane could be used as a solvent to dissolve EEPDMS while other organic liquids would be excluded due to their poor solubility and miscibility. However, it is a big obstacle for the hexane due to the large used amount in previous study [10]. By contrast, a relatively excellent thickening performance was illustrated by a small amount of Toluene as a solvent [14,38]. Thus, after, Toluene could be used as a solvent to measure the thickening property of EEPDMS.

### 3.3. Solubility and Phase Behavior of EEPDMS in Liquid CO_2_

In this paper, a visual method was employed and a windowed high-pressure cell with a variable volume used to observe the solubility and phase behavior of EEPDMS in Toluene/CO_2_ mixtures [30]. The detailed operating procedure, conducted at this high-pressure cell shown in Figure 2, was explained in our previous articles [18], and would not be described in detail. The results of phase behavior showed that the cloud point pressure was below 8 MPa with increasing temperatures in the range from 293 to 333 K and contents in the range of 1 to 3 wt.%. The fact that EEPDMS possessed a lower dissolved pressure in CO_2_ than that in other articles [10,14,19] suggested that EEPDMS was soluble in CO_2_ as easily as desired.

### 3.4. Apparent Viscosity and Rheology of the Thickened Liquid CO_2_

#### 3.4.1. Effect of Temperature on the Apparent Viscosity and Rheology

**Figure 4:**
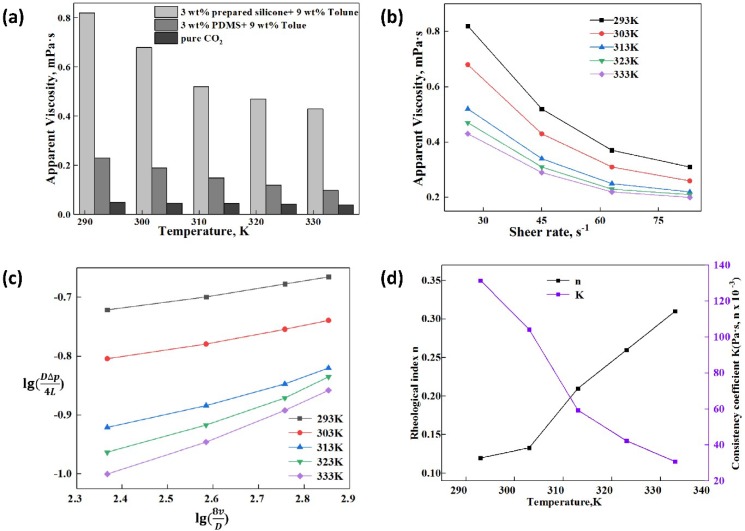
Effect of temperature on the apparent viscosity and rheology; (**a**) Temperature and apparent viscosity; (**b**) Shear rate and apparent viscosity; (**c**) Log*τ_w_* and Log(8*v*/*D*); (**d**) Rheological index, consistency coefficient and temperature.

The apparent viscosity and rheology of thickened liquid CO_2_ were influenced by many factors such as temperature, pressure, EEPDMS content (wt.%) and the shear rate. Figure 4 indicated the effect of temperature on the apparent viscosity and rheology of the thickened liquid CO_2_, and the pressure was identified as 8 MPa at 3 wt.% EEPDMS. In addition, this investigation showed the comparison of thickening performance between EEPDMS and polydimethylsiloxane (PDMS). In the first place, measurements carried out with EEPDMS resulted in the apparent viscosity of thickened CO_2_ three to four times higher that of PDMS. In comparison with PDMS, the CO_2_ viscosity increased evidently after adding EEPDMS into CO_2_, indicating that EEPDMS was provided with an excellent thickening performance than that of PDMS as desired. The results showed that the apparent viscosity of thickened CO_2_ decreased progressively with increasing the temperature. In comparison to previous research, there was a same trend between the apparent viscosity and temperature [9,10,14]. It was attributed to the effect of temperature on the space mesh structure of thickened CO_2_ [9]. A space mesh structure formed based on the C–H···O bond [2,39,40,41] existed between CO_2_ and Toluene and the interaction between Toluene and siloxane [2]. The apparent viscosity of thickened CO_2_ was closely related to the density of these space mesh structures, the bond length between molecules and winding intensity of space mesh. As temperature increases, these above bonds formed between molecules gradually break, this formed mesh structure is destroyed gradually owing to the increasing molecular activity. Meanwhile, the Arrhenius equation could correctly explain the influence of temperature on the apparent viscosity of thickened CO_2_ as shown in Equation (2) [40,42].

(2) μ=Avexp(EfRgT)

where *μ* is the fluid viscosity, *A_v_* is the pre-exponential factor, *E_f_* is the activation energy, *R_g_* is the molar gas constant. The temperature (*T*) was limited to a small range of 293 to 323 K. The migration activity of every molecule improved with the increase in the system temperature. The gradual broken space mesh structure caused decrease in the activation energy of system and flow resistance. The thickened CO_2_ illustrated a reduction in liquid viscosity [9,39,40]. It should be noted that the shear rate is between 0 and 100 s^−1^ mainly because the viscosity hardly changes when the shear rate is greater than 100 s^−1^, namely, the viscosity at a high shear rate bigger than 100 s^−1^ is stable.

Meanwhile, it could be seen from Figure 4c,d that the rheological index ‘n’ increased with the increase in the system temperature, which resulted from the apparent viscosity reduction. However, a decreasing trend contributed to the consistency coefficient K when the system temperature rose. The bigger the system temperature was, the stronger the non-Newtonian property became.

#### 3.4.2. Effect of Pressure on the Apparent Viscosity and Rheology

**Figure 5:**
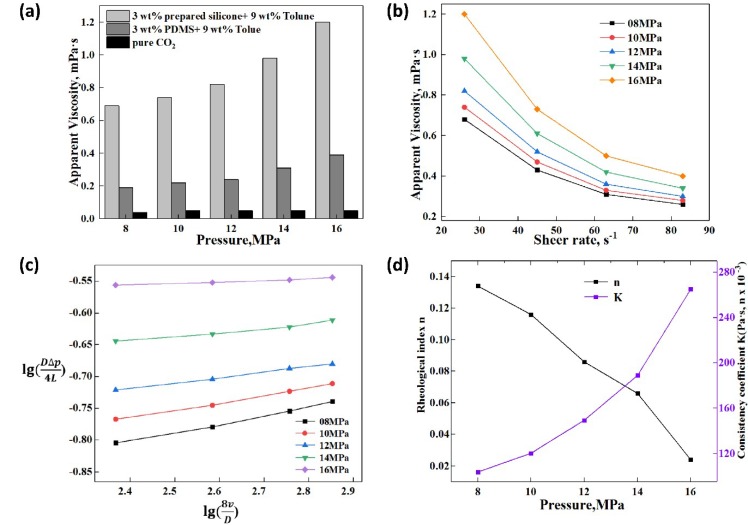
Effect of pressure on the apparent viscosity and rheology. (**a**) Temperature and apparent viscosity; (**b**) Shear rate and apparent viscosity; (**c**) Log*τ_w_* and Log(8*v*/*D*); (**d**) Rheological index, consistency coefficient and temperature.

It could be seen from Figure 5 that the relationship between the apparent viscosity of CO_2_, rheology and pressure was shown at the temperature of 303 K and 3 wt.% EEPDMS. An increase CO_2_ viscosity was seen with an increase in the pressure from 8 to 16 MPa, which was similar to previous research conclusions [9,10,14,19]. In comparison with EEPDMS, PDMS illustrated a poor thickening capability, which resulted from the influence of pressure on the mesh structure and the molecular spacing. The molecular spacing was shortened with increasing the pressure, and more compact mesh structures were formed at a higher pressure. The porosity of the microscopic mesh structure was significantly reduced to form a large flow resistance [9], which resulted in a slow increase in viscosity. Moreover, as the system pressure rose, these electron-donating groups such as epoxy ether group showed an enhanced solubility and interaction with CO_2_ [16,17], which contributed to the improvement in viscosity.

The effect of pressure on the apparent rheology of the liquid CO_2_ was displayed in Figure 5c,d. The consistency coefficient K built up over the increase in the pressure but the rheological index n rapidly reduced. When the pressure rose, the spatial distance between molecules was progressively shortened. The interaction among molecules was intensified [9]. The above reasons resulted in a denser mesh structure to improve the viscosity and rheology. In addition, Non-Newtonian property of this thickened liquid CO_2_ enhanced when the pressure rose. The effect of the pressure on the rheology of this thickened liquid CO_2_ will be lesser than that of the temperature depending on the rheological index n and consistency coefficient K in the Figure 4d and Figure 5d.

#### 3.4.3. Effect of Thickener Content on the Apparent Viscosity and Rheology

**Figure 6:**
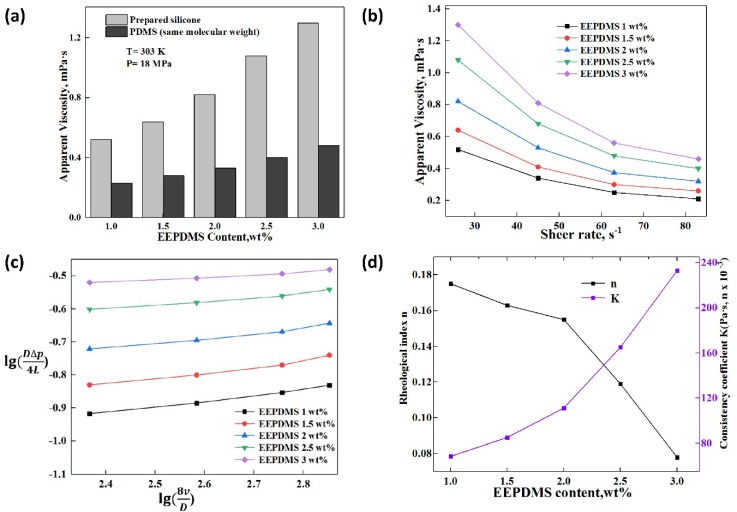
Effect of thickener content on the apparent viscosity and rheology. (**a**)Temperature and apparent viscosity; (**b**) Shear rate and apparent viscosity; (**c**) Log*τ_w_* and Log(8*v*/*D*); (**d**) Rheological index, consistency coefficient and temperature.

**Figure 7:**
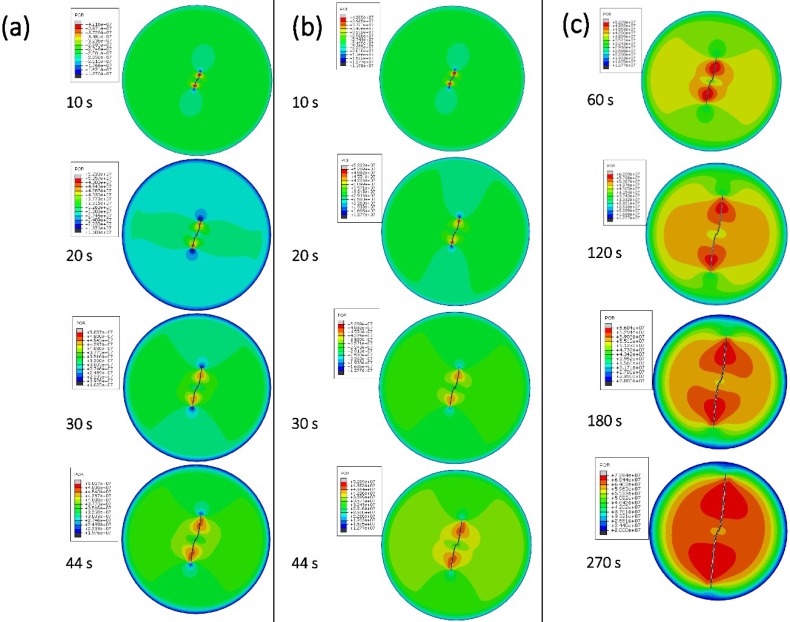
The distribution of the fracture morphology evolution. (**a**) Pure liquid CO_2_ (0.04 mPa·s, 0–44 s); (**b**) Thickened liquid CO_2_ (1.3 mPa·s, 0–44 s); (**c**) Thickened liquid CO_2_ (1.3 mPa·s, 0–270s).

This apparent viscosity indicated in Figure 6a and Figure 7b were tested at 303 K and 18 MPa, which could form a single-phase fluid. In addition to the thickening data of EEPDMS, Figure 6a also displays the apparent viscosity data for the pure CO_2_ and the thickened CO_2_ with the use of PDMS. The thickening performance as displayed in Figure 6a indicated differences between EEPDMS and PDMS. The CO_2_ viscosity became smaller for the PDMS compared with that of EEPDMS. This is attributed to the epoxy group and carbonyl group inside ester group which does not only assist EEPDMS to enhance the interaction between the thickener and CO_2_, but also improve the solubility and miscibility of EEPDMS in CO_2_ [16,17]. Meanwhile, EEPDMS content was significantly increased during the measurement, which caused further increase in the apparent viscosity. More compact mesh structure formed due to the increase of EEPDMS in CO_2_, which showed more chemical bonds among CO_2_, EEPDMS, and Toluene. Much force is required to break the mesh structure [9,40]. In this scenario, the apparent viscosity becomes greater with an increase in the EEPDMS content.

The slope of each line in Figure 6c presents the rheological index n under certain conditions [9,40]. As you can see by Figure 6c,d, the rheological index n decreased with increasing the EEPDMS content. However, the consistency coefficient presented a rising trend. The rheological index n under different EEPDMS content, reduced from 0.175 to 0.078, demonstrating that the non-Newtonian characteristic of the thickened liquid CO_2_ became greater as the thickener content increased [43].

#### 3.4.4. Effect of Shear Rate on the Apparent Viscosity and Rheology

Figure 4a and Figure 5a demonstrated that the apparent viscosity of pure CO_2_ did not change until EEPDMS or PDMS was added into it, which indicated that pure CO_2_ was a Newtonian fluid. By contrast, the thickened liquid CO_2_ with EEPDMS or PDMS showed a decreased curve with increasing the shear rate (see Figure 4b, Figure 5b and Figure 6b), which indicated that the thickened CO_2_ fracturing fluid was a power law fluid with the shear thinning property [9,14,39,40]. The effect of shear rate on the pure CO_2_ viscosity can be practically negligible and this can be attributed to the similarity in the properties between CO_2_ and Newtonian fluid [9]. Shear thinning property of thickened CO_2_ can be attributed to the space mesh structure destruction relating to gradually increased shearing force with increasing the shear rate.

There were many novelties of EEPDMS shown in this paper. The low dissolved pressure could allow EEPDMS to be used in reservoirs with a low pressure. Moreover, EEPDMS showed an excellent thickening performance. Doherty [10] also prepared the thickener with an excellent thickening performance but the huge measurement pressure and cosolvent amount (>40 wt.%) was a huge challenge that hindered the application of silicone polymer as a thickener in the CO_2_ fracturing.

### 3.5. Numerical Simulation

A shale reservoir model displayed in Figure 3, based on the basic physical parameter of shale reservoirs, was established to verify the fracturing property of thickened liquid CO_2_. It could be seen from Figure 7 that an obvious crack turning characteristic was both illustrated between the pure CO_2_ fracturing fluid and thickened CO_2_ fracturing fluid (The parameters required for fracturing fluid are shown in Table 3). These two fracturing fluids still showed obvious crack morphology differences.

**Figure 8:**
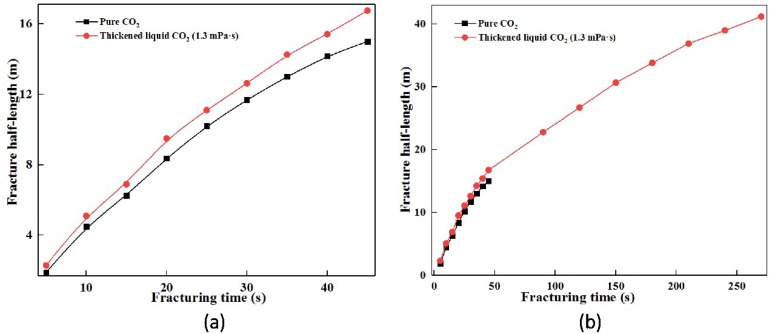
The effect of EEPDMS on the fracture half-length in CO_2_ fracturing operation. (**a**) 0–44 s; (**b**) 0–270 s.

The pure CO_2_ conducted a tardily propagated prefabricated perforation with the effect of the loading, and it eventually failed at 44 s. However, the excellent fracturing property was shown by the thickened CO_2_ fracturing fluid. Compare to the difference in crack propagation, a conclusion can be drawn that with the increase of CO_2_ viscosity, the fracture half-length increased dramatically (Figure 8). The fracture half-length of pure CO_2_ fracturing fluid was merely 15.5 m at 44 s, whereas the thickened liquid CO_2_ obtained a fracture half-length of 41.4 m at 270 s (Figure 8b). In addition, it could be seen from Figure 8a that the fracture half-length of thickened liquid CO_2_ (1.3 mPa·s) is slightly larger than that of the pure liquid CO_2_, and the fracture half-length of thickened liquid CO_2_ showed a growth trend. The formed actual fluid pressure (Pressure inside the crack) (see Figure 7) was considered to be the reason for the different fracture half-length during CO_2_ fracturing operation. The viscosity of fracturing fluid is an important variable that influences fracture propagation [44,45]. The higher the fluid viscosity the lower the local flow rate in the fracture and the higher the fluid pressure and breakdown pressure under the same injection rate [46,47]. Moreover, the initiation pressure increased with an increase in the fluid viscosity [48]. With increasing dynamic viscosity, the breakdown pressure increases significantly but the fracture initiation pressure increases only slightly [48]. However, it could be seen from Figure 7 that there is a significant increase in the fluid pressure (Pressure inside the crack) when the viscosity is increased. Compared to the liquid pressure of the low viscosity (<20 MPa at 44 s), the high fluid pressure (>29 MPa at 44 s) of the higher viscosity could break through the reservoir pressure. This breakdown pressure and initiation pressure can continue the fracturing operation. However, the liquid pressure of the low viscosity (Pure liquid CO_2_, <20 MPa at 44 s) could not eliminate the above three pressures, and the fracturing operation suspended.

## 4. Conclusions

The results showed that EEPDMS have a considerable influence on the CO_2_ viscosity. Compared with the pure CO_2_, EEPDMS addition was contribute to the interaction with CO_2_, generating more microscopic mesh structure, which could be used to explain the increase in CO_2_ viscosity. On the other hand, the influence of temperature, pressure, shear rate and EEPDMS content on the CO_2_ viscosity was inconsistent. As temperature or shear rate increased, the CO_2_ viscosity decreased. However, CO_2_ viscosity displayed a rising trend with increasing the pressure or EEPDMS content. These above trends were applicable not only to pure CO_2_ but also to thicken CO_2_. The mesh structure theory was important as it applied to explain the causes of viscosity changes by various factors. The established shale model described the fracturing effect of the thickened liquid CO_2_. In the future study, we could summarize the design principle of CO_2_ thickener to prepare a thickener with an excellent thickening CO_2_ performance which will be beneficial for the CO_2_ use in shale gas mining. The simulation results of CO_2_ fracturing in this article provided a theoretical basis for the application of CO_2_ fracturing technology, and we will do a deeper study of the CO_2_ fracturing experiment and give the data in a further paper.

## Figures and Tables

**Table 1:** The characteristic parameters and the initial conditions of shale reservoirs.

Parameter	Value	Parameter	Value
Elastic Modulus, *E*/GPa	20	Poisson’s ratio, *ν*	0.26
Minimum horizontal principal stress, *σ_h_*/MPa	31	Maximum horizontal principal stress, *σ_H_*/MPa	37
Tensile strength, *C*/MPa	3.5	Initial pore pressure, Pip/MPa	18
Initial porosity, *ϕ*/%	8	Permeability, *K*/m^2^	4 × 10^−17^
Injection rate, *Q*/(m^3^/min)	4	Total fracturing time, *T*/min	10
Leak-off coefficient	1 × 10^−14^	Viscosity, mPa·s	0.04 and 1.3

**Table 2:** Viscosity and Phase behavior of EEPDMS on Organic Liquids.

Solvent	EEPDMS Content (wt.%)	Solution State	283 K	293 K	303 K	313 K	323 K
Toluene	1	Clear, Viscous liquid	M	M	M	S	S
Hexane	1	Clear, Viscous liquid	M	M	M	S	S
Pentane	1	Clear, Viscous liquid	M	S	S	S	S
Nonanol	1	Turbid solution	I	I	I	I	S
Methanol	1	Turbid solution	I	I	I	I	I
Ethanol	1	Turbid solution	I	I	I	I	I
1,2-Propylene glycol	1	Turbid solution	I	I	I	I	I
Toluene	2	Clear, Viscous liquid	M	M	M	S	S
Hexane	2	Clear, Viscous liquid	M	M	M	S	S
Pentane	2	Clear, Viscous liquid	M	S	S	S	S
Nonanol	2	Turbid solution	I	I	I	I	I
Methanol	2	Turbid solution	I	I	I	I	I
Ethanol	2	Turbid solution	I	I	I	I	I
1,2-Propylene glycol	2	Turbid solution	I	I	I	I	I
Toluene	4	Clear, Viscous liquid	M	M	M	M	S
Hexane	4	Clear, Viscous liquid	M	M	M	M	S
Pentane	4	Clear, Viscous liquid	M	M	S	S	S
Nonanol	4	Turbid solution	I	I	I	I	I
Methanol	4	Turbid solution	I	I	I	I	I
Ethanol	4	Turbid solution	I	I	I	I	I
1,2-Propylene glycol	4	Turbid solution	I	I	I	I	I

* M: Medium viscosity (0.5–0.7 mPa·s); S: Slight viscosity (0.1–0.5 mPa·s); I: Insoluble.

**Table 3:** The Detailed parameters of fracturing fluids for numerical simulation in this paper.

Fluid type	Viscosity (mPa s)	Injection Rate (m^3^/min)	Thickener Content (wt.%)	Cosolvent Content (wt.%)
Pure liquid CO_2_	0.04	4	0	0
Thickened liquid CO_2_	1.30	4	3	9

## Supplementary Materials

The following are available online at https://www.mdpi.com/2073-4360/11/3/540/s1, Figure S1: FTIR spectra analysis of EEPDMS. Figure S2: 1H-NMR (CDCl3) spectra at 400 MHz of EEPDMS.

## Nomenclature

*σ_h_*	Minimum horizontal principal stress
*σ_v_*	Minimum vertical principal stress
*P_f_*	Injection pressure of CO_2_ fracturing fluid
