# Supplementary material for: Effect of a Modified Silicone as a Thickener on Rheology of Liquid CO2 and Its Fracturing Capacity
Source: Polymers (Basel). 2019 Mar 21;11(3):540. doi: 10.3390/polym11030540 (PMC6473470; doi:10.3390/polym11030540)
Supplement: Supplementary file 1 [file polymers-11-00540-s001.pdf]

Supplementary Material

Effect of a modified silicone as a thickener on the viscosity and rheology of liquid CO_2 and the evaluation of fracturing capacity

Qiang Li [1*], Yanling Wang [1, *], Qingchao Li [1], Fuling Wang [2], Hao Bai[1], Lin Yuan [1], Forson Kobina [1]

[1] College of Petroleum Engineering, China University of Petroleum (East China）, Qingdao, 266580, China; Email: wangyl@upc.edu.cn (Yanling Wang); B16020083@s.upc.edu.cn (Qiang Li) ; B16020053@s.upc.edu.cn (Qingchao Li); forsonkobina@ymail.com (Forson Kobina); 702076238@qq.com (Hao Bai); 1255163031@qq.com (Lin Yuan)

[2] College of Science, China University of Petroleum (East China）, Qingdao, 266580, China; Email: B18090002@s.upc.edu.cn (Fuling Wang)

* Correspondence: wangyl@upc.edu.cn (Yanling Wang); B16020083@s.upc.edu.cn (Qiang Li);

Table of contents

1 FTIR (EEPDMS) spectra S1

2 ^{1}H NMR spectroscopy S2

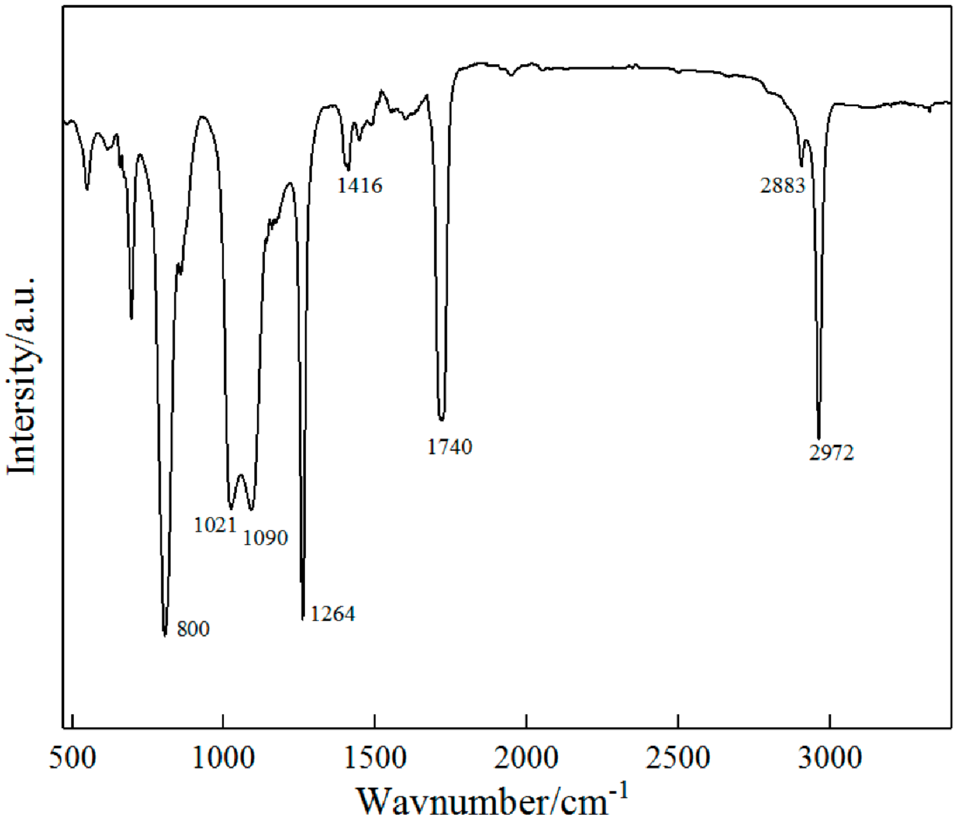

Figure S1. FTIR spectra analysis of EEPDMS

The synthesis and post-modifications of Polydimethylsiloxane, namely EEPDMS, was monitored by infrared spectroscopy (see Supplementary). It can be seen from the infrared spectroscopy that the sharp peak at 2972 cm^{-1} and 2883 cm^{-1} correspond to the C-H stretching vibration, and the peak at 1740 cm^{-1} can be attributable to the C=O bending vibration [S1]. The sharp peak observed 1416 cm^{-1} indicated the asymmetric vibration of Si-CH_3. In addition, the narrow peak of 1170 cm^{-1} (obstructed by the Si-O-Si peak at 1021-1200 cm^{-1}) and 1018 cm^{-1} indicated the stretching vibration of C-O-C group and the epoxy group respectively. The absorption band occurring between 1090±1020 cm^{-1} indicated the symmetrical stretching vibration of the Si-O-Si [S2]. Meanwhile, the peak at 1264 cm^{-1}, attributable to the symmetrical vibration of Si-CH_3 and CH_3, and the peak at 800 cm^{-1} confirmed the presence of the telescopic vibration of Si-C.

Reference:

[S1]: Gnanasambandam, R., Proctor, A. Determination of pectin degree of esterification by diffuse reflectance Fourier transform infrared spectroscopy. *Food. Chem*, **2000**, 68(3), 327-332.

[S2]: Li, Q.; Wang, Y.; Li, Q.; Foster, G.; Lei, C. Study on the optimization of silicone copolymer synthesis and the evaluation of its thickening performance. *RSC Adv*. **2018**, *8(16)*, 8770-8778.

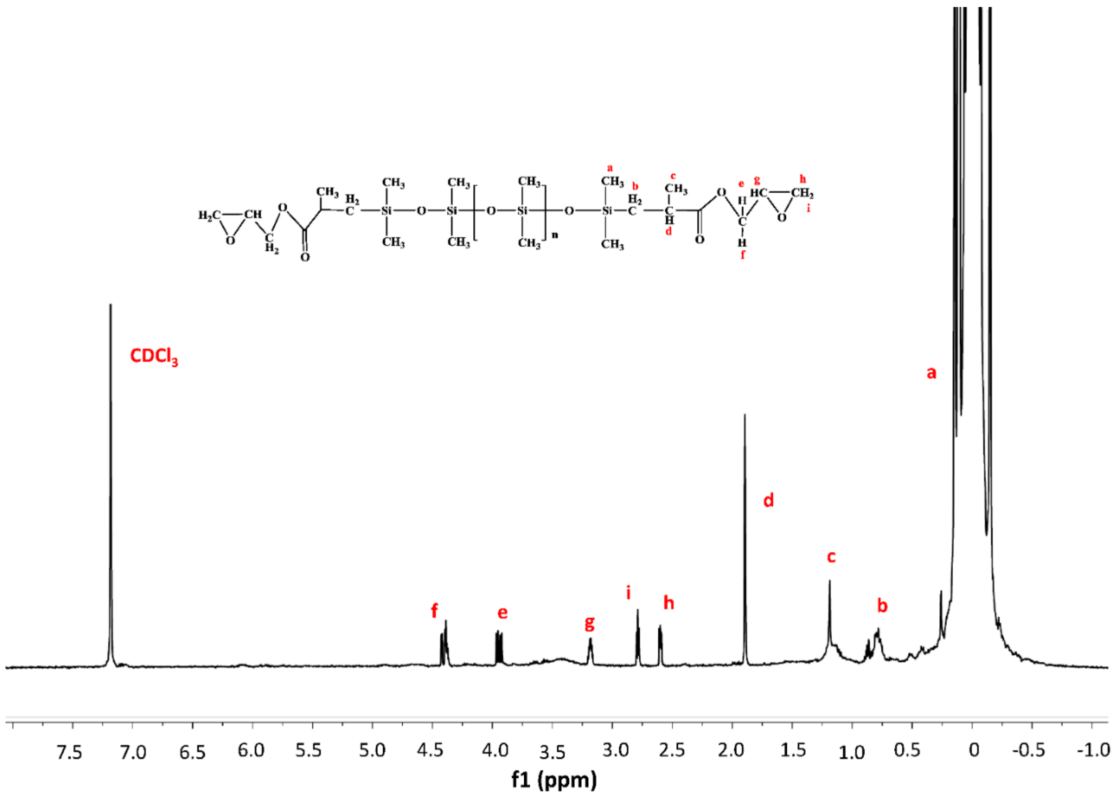

Figure S2. **^{1}H-NMR ($CDCl_3$) spectra at 400 MHz of EEPDMS**

Each hydrogen element of EEPDMS is labeled in Figure S2. It is clear that the chemical shift from 0.11 ppm to 0.28 ppm presented the H (a) of a methyl group, the methylene H connected to a silicon atom attributed to the 0.69 ppm (b). Meanwhile, the 2.54 ppm (h), 2.79 ppm (i) and 3.17 ppm (g) were considered to be the H of the homomethyl and methylene respectively. The chemical shift of Si-H does not appear in the range of 4.5 to 5.3 ppm [S3] and the chemical shift of the C=C bond do not occur in the range of 5.0 to 5.8 ppm [S4, S5]. The chemical shift at 1.19 ppm was the Hydrogen on the methyl (c) [S4] and the 1.89 ppm (d) presented the chemical shift of the C-H. In addition, the chemical shift at 3.86 ppm (e) and 4.34 ppm (f) was view as two hydrogens on the methylene group respectively.

Reference:

[S3] Berlekamp, U. H., Jutzi, P., Mix, A., Neumann, B., Stammler, H. G., Schoeller, W. W. Stabilization of Organosilicenium Ions by means of Intramolecular Coordination of O, S, or P Ligands. *Angew. Chem. Int. Edit*, ***1999***, *38*, 2048-2050.

[S4] van, Dijk-Wolthuis. W. N. E., Franssen, O., Talsma, H., van Steenbergen, M. J., Kettenes-van den, Bosch, J. J., Hennink, W. E. Synthesis, characterization, and polymerization of glycidyl methacrylate derivatized dextran. *Macromolecules*, ***1995***, *28(18)*, 6317-6322.

[S5] Virtanen, J., Tenhu. H. Studies on copolymerization of n-isopropylacrylamide and glycidyl methacrylate. *J. Polym. Sci. Pol. Chem*, ***2001***, *39(21)*, 3716-3725.
